# *E. coli* Is a Poor End-Product Criterion for Assessing the General Microbial Risk Posed From Consuming Norovirus Contaminated Shellfish

**DOI:** 10.3389/fmicb.2021.608888

**Published:** 2021-02-19

**Authors:** Jasmine H. Sharp, Katie Clements, Mallory Diggens, James E. McDonald, Shelagh K. Malham, Davey L. Jones

**Affiliations:** ^1^School of Ocean Sciences, Bangor University, Bangor, United Kingdom; ^2^School of Natural Sciences, Bangor University, Bangor, United Kingdom; ^3^UWA School of Agriculture and Environment, University of Western Australia, Perth, WA, Australia

**Keywords:** shellfish handling, mussel (*Mytilus edulis*), food safety, microbiological standard, risk assessment, STEC, norovirus

## Abstract

The fecal indicator organism (FIO) *Escherichia coli* is frequently used as a general indicator of sewage contamination and for evaluating the success of shellfish cleaning (depuration) processes. To evaluate the robustness of this approach, the accumulation, retention, and depuration of non-pathogenic *E. coli*, pathogenic *E. coli* O157:H7 and norovirus GII (NoV GII) RNA were evaluated using a combination of culture-based (*E. coli*) and molecular methods (*E. coli*, NoV GII) after exposure of mussels (*Mytilus edulis*) to water contaminated with human feces. We simulated water contamination after a point-source release from a combined sewer overflow (CSO) where untreated wastewater is released directly into the coastal zone. All three microbiological indicators accumulated rapidly in the mussels, reaching close to maximum concentration within 3 h of exposure, demonstrating that short CSO discharges pose an immediate threat to shellfish harvesting areas. Depuration (72 h) in clean water proved partially successful at removing both pathogenic and non-pathogenic *E. coli* from shellfish tissue, but failed to eradicate NoV GII RNA. We conclude that current EU standards for evaluating microbiological risk in shellfish are inadequate for protecting consumers against exposure to human norovirus GII found in polluted marine waters.

## Introduction

Globally, 18 million tons of marine molluscs are harvested each year with an estimated value of $35 billion, comprising 9% of the value of fisheries worldwide (Wijsman et al., 2019; FAO, 2020). In addition, shellfish consumption is becoming an increasingly important part of the human diet and is seen as an area for expansion and economic growth in many countries (Oehlenschlager, 2012; Silver, 2014; Bostock et al., 2016). Sustainable shellfish production, however, relies on the presence of clean water that is free from both organic and inorganic pollutants (Mohebbi-Nozar et al., 2014; Blanco, 2018; Enuneku et al., 2018). Microbiological contamination of molluscan shellfish can readily occur in areas where coastal waters are polluted with sewage effluent, and agricultural run-off, such that shellfish accumulate large amounts of bacterial or viral pathogens (Bosch et al., 1995; Burkhardt and Calci, 2000; Winterbourn et al., 2016). In many parts of the world, shellfish are grown in areas exposed to treated or untreated wastewater which may also contain human pathogens (e.g., Cryptosporidium, hepatitis A/E, norovirus; Razafimahefa et al., 2020). Therefore, raw and undercooked shellfish can act as vectors of infectious pathogens which can pose a serious risk to human health (Flannery et al., 2014; Santos-Ferreira et al., 2020).

Bacterial species are traditionally used as indicators of fecal contamination of agricultural products, shellfish and shellfish waters (Garcia et al., 2020). This has led to the formulation of legislation based on the measurement of fecal indicator bacteria (EU, 2020). For example, within the European Union (EU), shellfish harvesting areas are classified according to the concentration of fecal indicator bacteria (*Escherichia coli*) as designated in Annex II of EU Regulation 854/2004 (EU, 2004). However, studies have indicated that monitoring fecal indicator bacteria in shellfish may be a poor indicator of water pollution and the risk of human exposure to pathogens from consuming shellfish (Gerba et al., 1979; Romalde et al., 2002; Younger et al., 2018). While methods for the detection and quantification of pathogens (e.g., *Vibrio* spp. viruses) in shellfish exist, these have yet to be incorporated into EU legislation due to the lack of a robust evidence base and disagreements over what new standards should be introduced (Lees and Cen WG6 TAG4, 2010; Hassard et al., 2017; EFSA, 2019).

If shellfish are shown to contain high levels of *E. coli* (>230 CFU 100 g^–1^ shellfish flesh) then they are deemed unfit for human consumption (Seafish, 2020). At this point, depuration or relaying to a new area is required to reduce contamination levels without further treatment or processing (McLeod et al., 2017a). Depuration is the process of controlled purification in land-based tanks where shellfish are cleansed using flow-through or recirculating water (Richards, 1988). Despite this controlled cleansing, “zero risk” to human health is rarely achievable with some pathogens often proving difficult to eliminate (Hassard et al., 2017; Amoroso et al., 2020). Typically, however, conventional depuration systems are effective at greatly reducing *E. coli* from shellfish to a standard that complies with current legislation requirements (Muniain-Mujika et al., 2002; Nappier et al., 2008; Love et al., 2010). However, it is unclear whether conventional systems are able to successfully depurate new and emerging pathogens and current legislation is inadequate in this respect, to protect public health. For example, shellfish have recently been implicated in outbreaks of norovirus and hepatitis A, despite undergoing depuration in compliance with EU standards (Diez-Valcarce et al., 2012; Maunula and von Bonsdorff, 2014).

The majority of research on the effectiveness of depuration has been conducted using oysters, as these are usually eaten raw. Less research is available on the effectiveness of depuration on mussels, particularly *Mytilus edulis* (common mussel), with regard to depuration of bacteria and viruses from mixed contamination sources. The first aim of this study was therefore to compare the accumulation, retention, and depuration of *E. coli* with pathogenic *E. coli* O157:H7 and norovirus in a controlled environment over a short time period. The short (24 h), time period was chosen to simulate acute contamination, as observed during combined sewer overflow (CSO) discharges, or other sewage overflow events. These events are known to occur on a frequent basis throughout the UK (Mounce et al., 2014). The second aim of this study was to demonstrate the effectiveness of current UK depuration conditions at the relative removal efficiency of *E. coli* and norovirus RNA in individual and mixed contamination conditions. Previous studies have largely focused on individual microbial indicators however, it is not known whether they interact in terms of bioaccumulation and subsequent elimination.

## Materials and Methods

### Cell Culture and Viral Stocks

A human gut isolate of *E. coli* was obtained from NCIMB, Aberdeen, United Kingdom (NCIMB 12413) as a lyophilised culture (Ni et al., 2016). A human isolate of *E. coli* serotype O157:H7 verocytotoxin-negative NCTC 12900 was obtained from NCTC, Porton Down, United Kingdom as a lyophilised culture (Bezanson et al., 2012). Lyophilised cultures were resuspended in 0.5 ml nutrient broth (Oxoid Ltd., Basingstoke, United Kingdom), sub-cultured twice onto nutrient agar (Oxoid Ltd.) to single colonies. Overnight broth cultures were diluted to an OD_600_ value of 0.5; 50 ml of the diluted cultures were centrifuged at 4,000 *g* for 10 min and resuspended in 1/4-strength Ringers solution (Oxoid Ltd.). Bacterial cultures were quantified by dilution series plating onto selective media and incubating for 24 h at 37°C prior to enumeration: *E. coli*/coliform harlequin media #008 (Lab M Ltd., Bury, United Kingdom; Pitkanen et al., 2007; Clements et al., 2013) for generic *E. coli*, CT-SMAC media (Oxoid Ltd) for *E. coli* O157:H7 (Jordan and Maher, 2006). Toxigenic *E. coli* O157 was chosen based on its prevalence in shellfish both in the United Kingdom and elsewhere (MacRae et al., 2005; Matulkova et al., 2013; Prakasan et al., 2018), although it should be noted that disease outbreaks directly linked to consumption of *E. coli* O157 contaminated shellfish are extremely rare. In addition, where they have been linked to outbreaks of gastrointestinal illnesses other pathogens have also been present in shellfish (co-contaminants; e.g., *Vibrio parahaemolyticus*, *Vibrio albensis*, *Shigella flexneri*) (CDC, 2019; CDPH, 2019).

Human-derived norovirus genogroup II (NoV GII.4/2012; Sydney-2012 like variant) positive fecal material was kindly provided by Prof. Ian Goodfellow (University of Cambridge, United Kingdom). This material was aliquoted and quantified using RT-qPCR prior to use in bioaccumulation experiments. The virus was not purified from the fecal material prior to use, however, the solid material was removed by centrifugation. The fecal material contained no detectable *E. coli* O157. Norovirus was chosen as numerous human disease outbreaks have been directly linked with consumption of norovirus contaminated shellfish (Fusco et al., 2017; Hassard et al., 2017).

### Bioaccumulation in Shellfish

*E. coli* and *E. coli* O157:H7 were each inoculated to a final concentration of 5 × 10^6^ CFU per 100 ml in 30 l tanks containing aerated natural seawater. These high contamination levels reflect periods when raw untreated sewage is released into UK coastal waters (Campos et al., 2011). Norovirus-positive fecal material was resuspended in a solution of 10% PBS (8 × 10^9^ gc ml^–1^; gc, genome copies) and inoculated into separate identical 30 l tanks to a final concentration of 2.7 × 10^5^ gc ml^–1^. *E. coli*, *E. coli* O157:H7 and norovirus-positive fecal material in the same quantities were added to a third set of identical tanks. These concentrations reflect high levels of contamination when the risk of bioaccumulation from exposure to raw sewage is greatest (e.g., during a large norovirus outbreak)(Katayama and Vinjé, 2017; Farkas et al., 2018). All tanks were contained in a climate-controlled chamber at 12°C to represent typical UK coastal water conditions (annual mean 12.4°C, min 7°C, max 17°C; Morris et al., 2018). Fresh seawater was obtained from the nearby Menai Straits (53°13′18.7″’ N, 4°9′36.6″’W) and had a salinity of 34‰. The pH was maintained at 7.5–8.0 by daily monitoring with a pH probe. NH_4_^+^, NO_2_^–^ and NO_3_^–^ were measured colorimetrically every 2 days according to Jones and Willett (2006) with values ranging from 0 to 0.2 mg l^–1^. The O_2_ content of the water was measured daily and shown to be fully saturated using a dissolved O_2_ probe (Hanna Instruments Ltd., Leighton Buzzard, United Kingdom). The room was maintained with a low light intensity to promote shellfish activity (Lee et al., 2008) and was conformed to a microbiological biosafety level 2 containment facility.

Mussels (*Mytilus edulis*; 4–5 cm shell length) were collected from a Class B shellfish bed in the Menai Strait by a local commercial fishing company. This site has a previous history of microbiological contamination of mussels linked to major foodborne disease outbreaks (Boxman et al., 2016). This is linked to the episodic release of untreated sewage into shellfish harvesting waters from the main wastewater treatment plant that serves the city of Bangor and surrounding region and also from network pumping stations. The release of untreated sewage is often highly episodic and leads to high spatial and temporal fluctuations in *E. coli* loadings in shellfish (Clements, 2013).

After immediate transfer to the laboratory, the detritus on the shell surface was removed (e.g., barnacles, tubeworm) and the shells briefly cleaned using sterilized seawater (Clements et al., 2013). Any non-viable mussels were discarded (i.e., damaged shells). The mussels (*n* = 20) were laid in a monolayer in each tank containing viral and bacterial contaminants. Replicate (*n* = 3) batches of mussels (*n* = 16 batch^–1^) were removed from individual tanks every 3 h over a 24 h period for bacterial and viral analysis. Control batches were also removed at 0 h to assess background contaminant levels.

### Shellfish Depuration

After the 24 h accumulation period, the mussels in the remaining tanks were surface cleaned using a multi-agent disinfectant [1% (v/v) Virkon^®^; Thermo Fisher Scientific United Kingdom Ltd., Loughborough, United Kingdom; Hernandez et al., 2000] and placed into experimental depuration systems consisting of 50 l tanks containing fresh recirculating seawater (15 l min^–1^) equipped with UV disinfection treatment (Vecton V2 600; TMC Ltd., London, United Kingdom) in the recirculation line. The flow rate was above the minimum industry guidelines (2 complete changes h^–1^; FSS, 2009). The depth of the water above the mussels (8 cm) reflected recommendations for commercial indoor depuration facilities (FSS, 2009). As single layer of shells was used to maximize water circulation. The UV treatment (25 W) also reflects that in commercial depuration units (Sunnotel et al., 2007; FAO, 2008). Mussels were retained at low density (ca. 6 kg m^–2^) in these conditions for 5 days at 12°C (industry minimum temperature for depuration is 5°C with maximum shellfish densities of 50 kg m^–2^; FSS, 2009; McLeod et al., 2017b). Water parameters, including salinity, pH, NH_4_^+^, NO_3_^–^, NO_2_^–^, O_2_ and temperature were monitored throughout, and maintained as described above. Due to the low shellfish density, at no time in the experiment did any of the water quality parameters fall close to the recommended minimum standard levels (McLeod et al., 2017b). Although higher water temperatures may facilitate the depuration of microbial contaminants, these were not used as it does not reflect current practice within the region (McLeod et al., 2017b). Vibrations in the facility were kept to a minimum and no shells failed to open (Younger et al., 2020). Replicate (*n* = 9) batches of mussels (*n* = 16 batch^–1^) were removed at time 3, 6, 9, 12, 18, 24, 36, 48, and 72 h for bacterial and viral analysis.

### Bacterial Enumeration

A sub-sample (*n* = 6) of the recovered mussels were surface sterilized (as described above), shucked and the tissues homogenized in a sterile blender with stainless steel blades for 60 s. PBS dilutions of homogenized pooled shellfish were assayed for *E. coli* and *E. coli* O157:H7 by dilution plating onto selective media as described previously. Microbial numbers were expressed as CFU per 100 g of shellfish flesh. At each accumulation time period, 50 ml water from each tank was retained for bacterial enumeration. Bacteria were enumerated by serial dilution on the same selective media as per shellfish. Bacteria were also enumerated from water at selected time points during depuration to confirm that shellfish were not re-exposed to contamination (data not presented).

### DNA and RNA Recovery

Viral and bacterial RNA/DNA recovery from shellfish was carried out according to the ISO 15216-1 method with very minor modifications (ISO, 2017). Mussels (*n* = 10) were surface sterilized, shucked and the digestive tissues removed and pooled to obtain a final weight of 2 g digestive tissue per replicate sample. Remaining tissues and intravalvular fluid were pooled in the same way to obtain 2 g non-digestive tissue per replicate. Mengovirus vMC_0_ was used as an extraction control (Winterbourn et al., 2016). The positive controls were derived from homogenates prepared as per the samples but after addition of 1 Lenticule^®^ disc of Norovirus Reference Material (Public Health England, London, United Kingdom). Tissues were macerated and homogenized 1:1 (w/v) in proteinase K solution (100 μg ml^–1^) to a paste-like consistency prior to shaking incubation (37°C, 300 rev min^–1^) for 1 h. Digested homogenates were then secondary incubated at 60°C for 15 min, prior to centrifugation (3,000 *g*, 5 min) and recovering the supernatant. Viral RNA and DNA was extracted from each homogenate using NucliSENS^®^ extraction kits and a MiniMag apparatus (BioMérieux, Craponne, France) from a 500 μl sample volume according to the manufacturer’s protocol with process controls as performed in Lowther et al. (2012) and Winterbourn et al. (2016). RNA/DNA was eluted in RNase-free sterile water and stored at −80°C prior to analysis.

Viral RNA was recovered from water by filtration of 500 ml following the addition of 25 mM MgCl_2_ at pH 4–6 (Lukasik et al., 2000). Viruses were filtered onto 0.45 μm cellulose nitrate filters (Sartorius AG, Göttingen, Germany). RNA was recovered from filter membranes using the standard phenol-chloroform method described by Griffiths et al. (2000).

### Real Time RT-PCR (RT-qPCR) and Real Time PCR (qPCR)

To evaluate *E. coli* accumulation in different parts of the mussel, a qPCR approach was used. Plate counting was not done on these samples as it was logistically impossible to achieve this within the intensive 3 h sampling periods (i.e., only total *E. coli* in the mussel tissue was evaluated using standard plate counting).

RT-qPCR for norovirus GI, norovirus GII and Mengovirus (extraction control) and qPCR for *E. coli* and *E. coli* O157:H7 was performed using a QuantStudio 6 thermocycler (Applied Biosystems, Foster City, CA, United States). For both *E. coli* strains, genesig primer/probe proprietary detection kits were utilized (PrimerDesign Ltd., Southampton, United Kingdom) with PrecisionPlus mastermix (PrimerDesign Ltd.) in a total volume of 15 μl, using 5 μl extracted DNA. For norovirus GI and GII, RNA Ultrasense qRT-PCR mix was used with a total volume of 25 μl, using 5 μl (10 ng) extracted RNA. Five hundred nanometer forward primers, IFRGI or IFRGII, were paired with 900 nM reverse primer, NEDG1 or COG2R, and 250 nM probe, TM9GI or IFRG2, as described in Lowther et al. (2012). Negative (water) and positive (positive material) qPCR controls were included in each qPCR/RT qPCR run. The presence of PCR inhibitors was tested using external process controls (mengovirus) or standard positive material for each of the tested pathogens (Lees and Cen WG6 TAG4, 2010; Winterbourn et al., 2016; Farkas et al., 2018). Quantification was estimated by standard curves constructed using serial dilutions of the appropriate RNA or DNA, plotting genome copy number against Cq values and expressed as genome copies per gram of tissue (gc g^–1^). The limit of quantification was 1 × 10^2^ gc g^–1^ and the limit of detection was 40 gc g^–1^ (Winterbourn et al., 2016).

### Statistical Analysis

Data were analyzed using PASW Statistics v18 (IBM Corp., Armonk, NY) and Minitab v18 (Minitab Inc., State College, PA). Normality was assessed using a one sample Kolmogorov–Smirnov test (*P* ≥ 0.05). Data which did not demonstrate normal distributions were log_10_ transformed. Temporal data were analyzed by two-way analysis of variance (ANOVA) and least significant difference (LSD) *Post-hoc* test using *P* < 0.05 as the cut-off for statistical significance.

## Results

### Accumulation of Pathogenic and Non-pathogenic *E. coli* in Mussels

**FIGURE 1:**
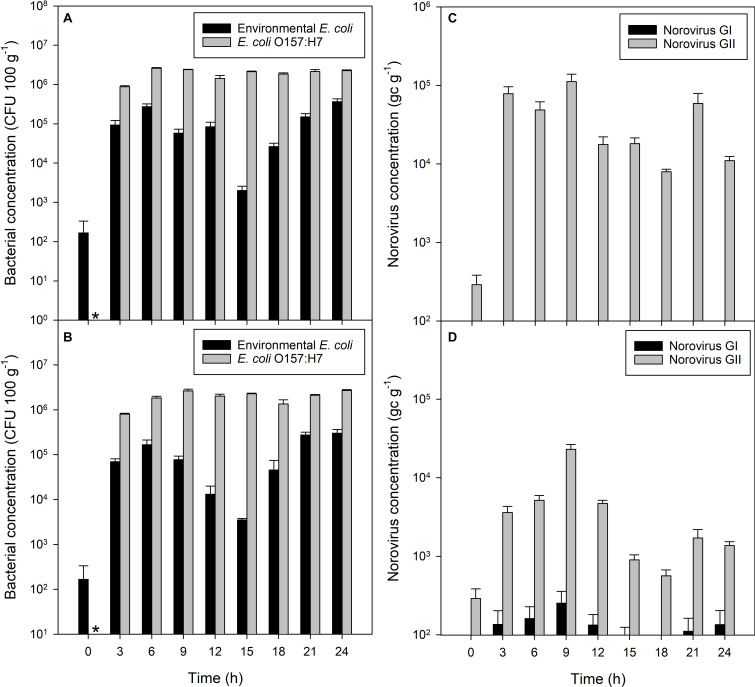
Accumulation of bacterial and viral contaminants in mussel flesh over 24 h of exposure to fecally contaminated seawater. Concentration in mussel flesh of **(A)** human gut-derived (non-pathogenic) *E. coli* and human-pathogenic *E. coli* O157:H7 in bacterial-only contaminated water, **(B)** human gut derived *E. coli* and *E. coli* O157:H7 in mussels exposed to mixed norovirus and *E. coli* contamination in the water, **(C)** norovirus GI and GII when exposed to viral-only contaminated water, and **(D)** norovirus GI and GII in mussels exposed to mixed norovirus and *E. coli* contaminated water. Values represent means ± SEM (*n* = 3). The asterisk indicates no *E. coli* O157:H7 in the mussels prior to contamination.

Non-pathogenic *E. coli* accumulated in shellfish tissue from an initial mean concentration of 166 CFU to 1 × 10^5^ CFU 100 g^–1^ within 3 h of exposure to water containing 5 × 10^6^ CFU *E. coli* 100 ml^–1^ seawater (*P* = 0.030; Figure 1A). This increased further to 3 × 10^5^ CFU 100 g^–1^ flesh after 6 h of exposure (*P* = 0.004). Non-pathogenic *E. coli* remained at this concentration in the mussel flesh, 1 log_10_ lower than the contamination of the seawater, until the end of the 24 h contamination exposure period. *E. coli* O157:H7 accumulated in the mussel flesh from an initial starting concentration of zero to approximately 10^6^ CFU 100 g^–1^ flesh within the first 3 h when exposed to an initial *E. coli* O157:H7 concentration in the seawater of 5 × 10^6^ CFU 100 ml^–1^ (*P* = 0.003; Figure 1A), increasing to 2 × 10^6^ CFU 100 g^–1^ flesh after 6 h, similar to levels of *E. coli* O157:H7 in the surrounding water.

Higher concentrations of *E. coli* O157:H7 in mussel flesh were observed than those of non-pathogenic *E. coli* in the same mussels, despite similar initial seawater concentrations (*P* < 0.001; Figure 1A). The concentration of *E. coli* O157:H7 was observed to be approximately 1 log_10_ higher than *E. coli* at all time periods during the exposure period.

**FIGURE 2:**
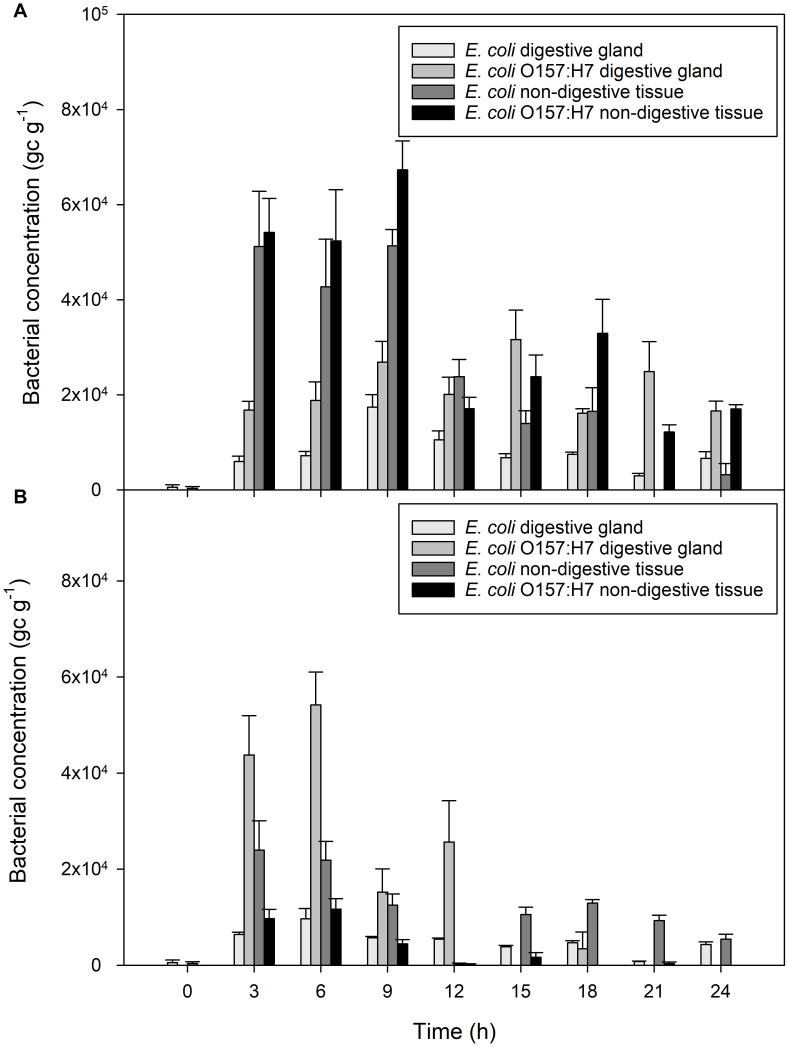
Accumulation of bacterial contaminants in different parts of the mussel over 24 h of exposure to fecally contaminated seawater **(A)** *E. coli* and *E. coli* O157:H7 in mussel digestive tissue or general mussel tissue when exposed to bacterial-only contaminated water, **(B)** *E. coli* and *E. coli* O157:H7 in digestive tissue and non-digestive material of mussels exposed to water containing a mixture of viral and bacterial contamination. Values represent means ± SEM (*n* = 9).

Bacterial contamination in the digestive tissue was compared to contamination in the general mussel tissue using qPCR (Figure 2A). Both non-pathogenic and pathogenic *E. coli* were measured in higher quantities in general (non-digestive) mussel flesh, compared to concentrations in the digestive tissue of the same mussel (both *P* < 0.001). The extent of this difference varied according to time since initial exposure (*P* < 0.001). After 3 h of exposure, non-digestive tissue contained 9 times more *E. coli* than digestive tissue, and 3.5 times more *E. coli* O157:H7 than the digestive tissue. Toward the end of the accumulation period, this ratio decreased. For example, after 18 h of exposure, non-digestive material contained twice the concentration of *E. coli* and *E. coli* O157:H7 than digestive tissue.

Endogenous norovirus strains GI and GII (i.e., that naturally present in the mussels before the experiments started) were measured in the mussels exposed only to the two *E. coli* cultures (i.e., no additional norovirus contamination). Overall, the initial concentrations were low (290 gc g^–1^) and no further norovirus GI or GII accumulated during the 24 h exposure period to the *E. coli*-only cultures (*P* = 0.975 and *P* = 0.136 for GI and GII, respectively).

### Accumulation of Norovirus in Mussels From Viral-Only Contaminated Waters

Norovirus GII concentration in the mussel flesh reached 7.8 × 10^4^ gc g^–1^ from an initial concentration of 290 gc g^–1^ within the first 3 h (*P* < 0.001; Figure 1C). A maximum concentration of norovirus GII of 1.1 × 10^5^ gc g^–1^ was reached within 9 h. Levels of norovirus GI did not change during the experimental period (*P* = 0.574). *E. coli* and *E. coli* O157:H7 in mussels was monitored, but remained unchanged throughout the 24 h exposure period (data not shown).

### Accumulation of *E. coli* and Norovirus From Co-contaminated Waters

When mussels were exposed to a mixed contamination of norovirus stool sample, non-pathogenic *E. coli* and *E. coli* O157:H7, uptake of pathogenic and non-pathogenic *E. coli* occurred rapidly (Figure 1B). The concentration of non-pathogenic *E. coli* in the mussel flesh increased from 170 CFU 100 g^–1^ to 7 × 10^4^ CFU 100 g^–1^ flesh within the first 3 h (*P* < 0.003), with a final concentration of 3 × 10^5^ CFU 100 g^–1^ flesh after 24 h of exposure (*P* < 0.008; Figure 1B). *E. coli* O157:H7 was undetected via colony counts at the start of the accumulation period, and increased to 8 × 10^5^ CFU 100 g^–1^ flesh within the first 3 h, increasing further to a maximum 2.7 × 10^6^ CFU 100 g^–1^ flesh after 9 h until the end of the 24 h contamination exposure period (Figure 1B). The initial concentration of pathogenic and non-pathogenic *E. coli* in seawater was 2 × 10^7^ CFU 100 ml^–1^. At all time periods, the level of *E. coli* O157:H7 contamination in the mussel flesh was 1 log_10_ higher than the contamination of the same mussels by non-pathogenic *E. coli* (*P* < 0.001).

Mussel digestive tissue contained greater quantities of *E. coli* O157:H7 than non-digestive tissue from the same mussels over the course of the experiment (*P* < 0.001). After 3 h of exposure, digestive tissue contained an average of 4.4 × 10^4^ gc g^–1^ *E. coli* O157:H7, compared to 9.7 × 10^3^ gc g^–1^ in non-digestive tissue, a fourfold increase in the tissue compared to non-digestive material (Figure 2B). In contrast, non-digestive tissue contained greater quantities of non-pathogenic *E. coli* than digestive mussel tissue, during all periods of exposure (*P* < 0.001; Figure 2B).

Very little norovirus GI was detected in the mussels (117 ± 21 gc g^–1^) during the experiment and these levels did not change significantly over time (*P* = 0.329). Norovirus GII concentrations in mussel digestive tissue increased from 290 to 3.6 × 10^3^ gc g^–1^ during the first 3 h of exposure (*P* = 0.012; Figure 1D). Contamination in the mussel digestive tissue varied between 2.3 × 10^5^ and 5.2 × 10^2^ gc g^–1^ over the 24 h exposure period, which is consistent with the level of contamination in the mussels exposed to norovirus only. A small difference in norovirus concentration between mussels exposed to virus only or mixed contamination was observed, with slightly less present in the mixed contamination (GI, *P* > 0.004; GII, *P* < 0.016). This level of reduction did not differ between genogroups (*P* = 0.143).

### Depuration of Pathogenic and Non-pathogenic *E. coli* in Bacterial-Only Contamination

**FIGURE 3:**
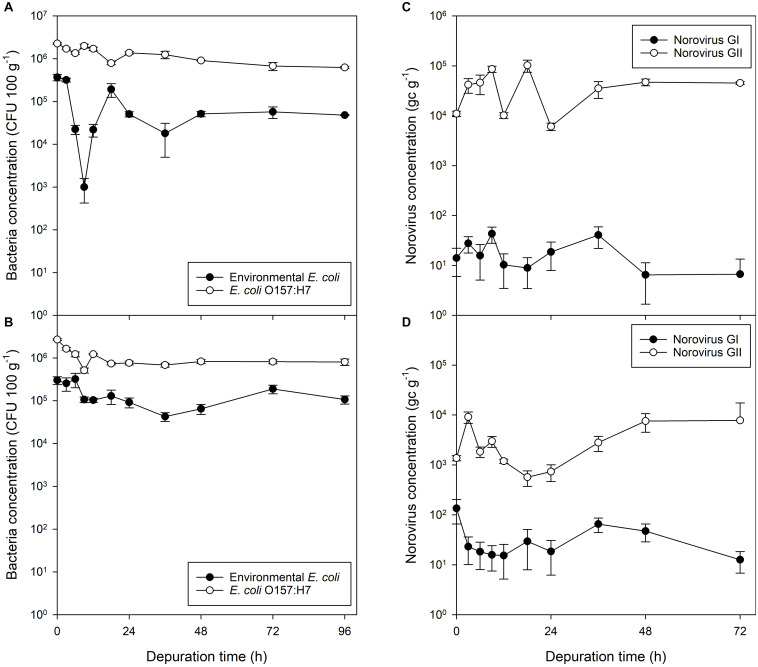
Bacterial and viral contaminants in mussel flesh over a 96 h depuration period after exposure to fecally contaminated seawater. Concentration in mussel flesh of **(A)** human gut-derived (non-pathogenic) *E. coli* and human-pathogenic *E. coli* O157:H7 previously exposed to bacterial-only contaminated water, **(B)** human gut-derived *E. coli* and *E. coli* O157:H7 in mussels exposed to mixed norovirus and *E. coli* contaminated water, **(C)** norovirus GI and GII when exposed to viral-only contaminated water, and **(D)** norovirus GI and GII in mussels previously exposed to water co-contaminated with norovirus and *E. coli*. Values represent means ± SEM (*n* = 3).

Eighty six percent of the non-pathogenic *E. coli* was depurated after 24 h. Following this, a slight reduction was also observed between 24 and 36 h. At the end of the 96 h depuration period, the reduction in non-pathogenic *E. coli* equated to a 87% decrease in concentration in comparison to that present at the start (*P* = 0.007). In comparison, 40% of the *E. coli* O157:H7 were depurated within 24 h, with a 48% reduction observed within 36 h. A 72% reduction in *E. coli* O157:H7 in mussel flesh was achieved in a 96 h period (*P* < 0.001; Figure 3A).

**FIGURE 4:**
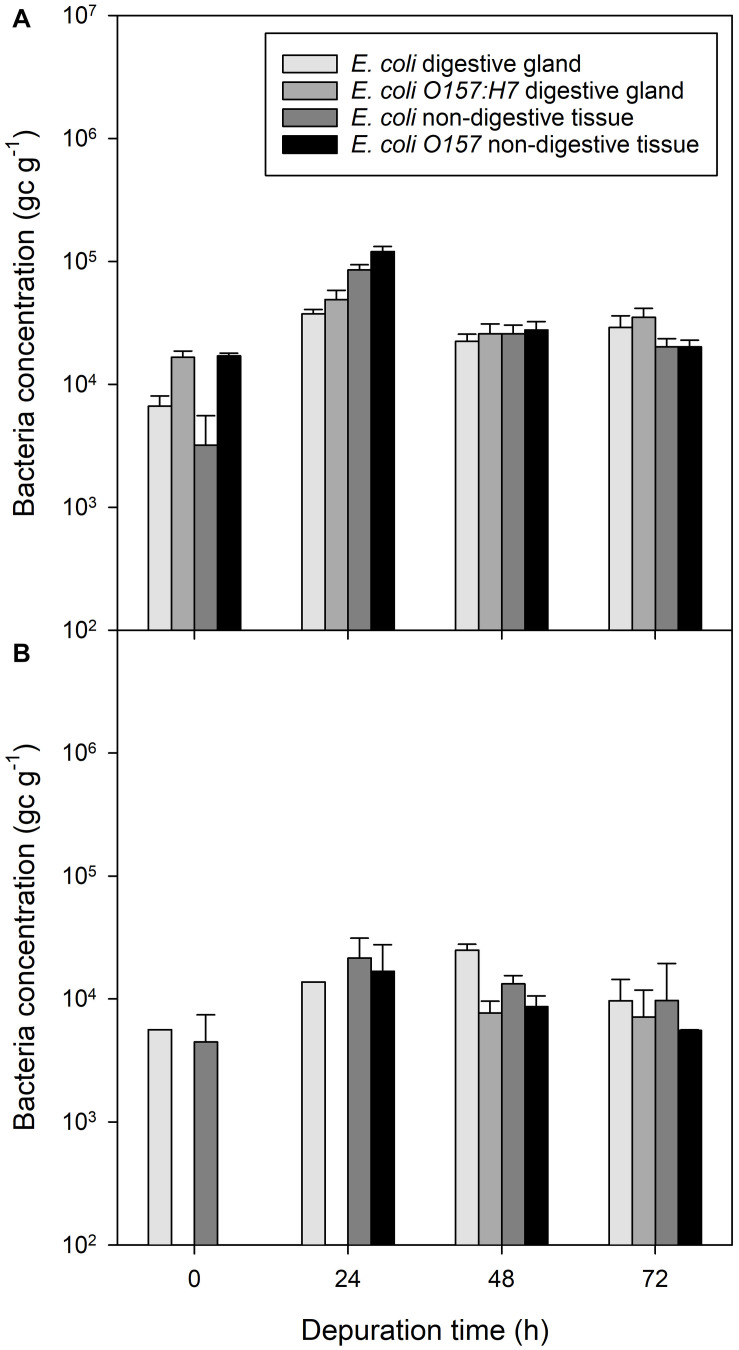
Presence of bacterial contaminants in different mussel tissues over a 96 h depuration period after exposure to fecally contaminated seawater. **(A)** *E. coli* and *E. coli* O157:H7 in mussel digestive tissue or general mussel tissue when previously exposed to bacterial-only contaminated water, **(B)** *E. coli* and *E. coli* O157:H7 in digestive tissue and non-digestive material of mussels previously exposed to water co-contaminated with norovirus and *E. coli*. Values represent means ± SEM (*n* = 9). The legend is the same for both panels.

During depuration, non-digestive tissue contained a lower concentration of *E. coli* and *E. coli* O157:H7 than digestive tissue from the same mussels between the start of depuration and 24 h into the depuration process (*P* < 0.01; Figure 4A). After 48 h of depuration, the concentration of bacterial DNA for both non-pathogenic *E. coli* and pathogenic *E. coli* O157:H7 was roughly equivalent between digestive tissue and non-digestive tissue (*P* = 0.586 and *P* = 0.803, respectively).

### Depuration of Norovirus From Mussels Experimentally Exposed to Norovirus-Only Contamination

Mussels experimentally exposed to norovirus contaminated material for 24 h demonstrated no decline in contamination of either norovirus GI or GII during the 72 h depuration period (Figure 3C). Norovirus GI contamination was at the limit of detection (average across all depuration samples, 19 ± 5 gc g^–1^), as the stool sample used during accumulation contained only norovirus GII. From the initial low level of norovirus GI contamination there was no change in concentration of GI in the mussel digestive tissue (*P* = 0.510). Norovirus GII in the digestive tissue continued to increase during the initial depuration period, for the first 18 h of depuration, to a concentration significantly higher than at the start of depuration (*P* = 0.030). During the 72 h depuration, the total observed decrease in norovirus GII from the start of depuration was 29%, however, this did not prove statistically significant (*P* = 0.363). The final norovirus concentration in the mussels was 4.5 × 10^4^ gc g^–1^.

### Depuration of Norovirus and *E. coli* From Mussels Co-contaminated With Faecally-Derived Bacteria and Viruses

Mussels exposed to mixed microbial contamination depurated 70% of their non-pathogenic *E. coli* during the first 24 h of depuration (*P* = 0.035), with little further loss seen after this time (Figure 3B). *E. coli* O157:H7 decreased by a similar proportion (71%; *P* < 0.001) over 24 h, but there was no subsequent decrease in *E. coli* O157:H7 between 24 and 96 h (*P* = 0.808; Figure 3B).

Mussels contaminated with a mixed culture of *E. coli*, *E. coli* O157:H7 and norovirus GII were again found to contain levels of norovirus GI (38 ± 12 gc g^–1^) which did not change during the 72 h depuration period (*P* = 0.152; Figure 3D). Similarly, the levels of norovirus GII did not change during the first 72 h of depuration (*P* = 0.087; Figure 3D). Norovirus GII was detected at similar concentrations at the start of depuration for all time points. The final norovirus concentration in the mussels was 7.8 × 10^3^ gc g^–1^.

In mussels co-exposed to *E. coli*, *E. coli* O157:H7 and norovirus GII, the concentrations of DNA associated with *E. coli* in the digestive tissue were observed to be similar to *E. coli* concentration in the non-digestive tissue at the start of depuration (Figure 4B). Non-pathogenic *E. coli* in the non-digestive tissue increased for the first 24 h of depuration, but the ratio of *E. coli* in the digestive tissue to non-digestive tissue was lower than observed in the *E. coli-*only exposed mussels at a ratio of approximately 1:2.

## Discussion

### Accumulation of *E. coli* in Mussels

Human pathogenic *E. coli* O157:H7 accumulated to a higher concentration in mussel flesh compared to the non-pathogenic *E. coli* strain. Despite similar initial concentrations in the seawater within the experimental exposure system, mussels accumulated *E. coli* O157:H7 2–10 times more effectively than non-pathogenic *E. coli* in the same tank. This occurred in both mussels exposed only to *E. coli*, and mussels co-exposed to both *E. coli* and norovirus. Mussels were found to accumulate non-pathogenic *E. coli* to a lower final concentration than that found in the surrounding water, whilst *E. coli* O157:H7 accumulated to higher concentrations in the shellfish than in the surrounding water. This suggests active accumulation of *E. coli* O157:H7, which may be due to differential binding of *E. coli* O157:H7 in the digestive tissue of the mussels. This differential accumulation is also mirrored in *Vibrio cholera* and *V. parahaemolyticus* accumulation in mussels and oysters (Collin et al., 2012; Aagesen et al., 2018). This also has parallels with humans where *E. coli* O157:H7 is known to cause disease in humans via preferential binding to the epithelial cells in the gut due to its high abundance of adhesins and fimbriae (Torres et al., 2005; Vidal et al., 2007). Our result is also consistent with the findings that depuration has a major effect on the bacterial diversity within mussel tissues, preferentially retaining some bacteria at the expense of others (Rubiolo et al., 2018).

Both types of *E. coli* were found to accumulate in the shellfish within the first 3 h of exposure, in agreement with previous studies of the uptake of *E. coli* and *Vibrio* spp. by mussels and other shellfish (Ho and Tam, 2000; McGhee et al., 2008; Herrfurth et al., 2013). In contrast, some other studies have shown *E. coli* not to be bioaccumulated in shellfish (e.g., *Crassostrea virginica*) beyond the level of water contamination (Burkhardt and Calci, 2000). This concurs with Polo et al. (2014b) and Younger et al. (2020) who suggest that viral uptake and removal rates from one species cannot be readily translated to another. In the case of shellfish beds becoming exposed to *E. coli*, we conclude that contamination will affect the *E. coli* loadings in the shellfish within the first 3 h of any spillage, which is critical when considering the impact of CSO discharges on shellfish (Shibata et al., 2014). However, it should be noted that pulses of sewage contaminated water may take many days to disperse in coastal environments depending on the prevailing currents (Robins et al., 2019). Our data show that environmental *E. coli* will accumulate to a concentration lower than the contaminating seawater. However, as *E. coli* O157:H7 is accumulated to a level equivalent to the surrounding seawater, *E. coli* O157:H7 contamination into the water can represent a much greater risk to surrounding shellfish beds. We also acknowledge that we only used two strains of *E. coli*. It is known that the human gut microbiome may contain many strains of *E. coli* which will ultimately enter the environment via wastewater discharge (Richter et al., 2018) and thus potentially accumulate in mussels to different extents (Oliveira et al., 2020). This work could therefore be further extended to investigate the abundance of different *E. coli* strains present in the mussel microbiome after the discharge of sewage into the marine zone.

### Norovirus Accumulation in Mussels

Similar to the fecal coliforms, norovirus GII was also found to accumulate in shellfish digestive tissue within the first 3 h of exposure, in agreement with previous studies demonstrating that norovirus accumulates in oysters within 4 h of exposure (Schwab et al., 1998). The findings from this study suggest that after an initial spike in norovirus GII, the concentration of GII in the digestive tissue decreases slightly over the subsequent 6–12 h. This is most likely due to progressive loss of viral integrity due to enzymatic action within the mussel’s digestive gland (Molloy et al., 2014; Kim et al., 2017).

### *E. coli* Depuration From Mussels

In this study, some of the non-pathogenic *E. coli* were removed by depuration within the 42 h time period typically used for commercial depuration in the UK. From high contamination levels, *E. coli* levels were successfully depurated by ca. 90%. However, from these high contamination levels a 90% decrease still represents a bacterial contamination level significantly greater than required under EU legislation for end-point product testing (230 CFU *E. coli* 100 g^–1^) (EU, 2004; FSS, 2009). Similarly, the elimination of non-pathogenic *E. coli* from the mussel flesh during the standard depuration period was similarly poor. We note, however, that the levels of *E. coli* O157 are reflective of highly sewage polluted waters and the depuration kinetics at low contamination levels requires further work. The same argument follows for Norovirus below.

There are numerous reports of Shiga-toxin-producing *Escherichia coli* (STEC) contamination of shellfish (Bennani et al., 2011; Baliere et al., 2015), however, surprisingly these have yet to be linked to actual shellfish-borne food poisoning outbreaks. Non-pathogenic *E. coli* depurated slightly faster in comparison to *E. coli* O157:H7. In the event of *E. coli* O157:H7 contamination in shellfish, depuration is likely to remove the non-pathogenic *E. coli* disproportionately to the more hazardous *E. coli* O157:H7. End-product testing of shellfish for *E. coli* will also detect the presence of *E. coli* O157:H7. However, the infectious dose of *E. coli* O157 serotypes has been shown to be less than 70 CFU person^–1^ (Tuttle et al., 1999) and is estimated by Public Health England to be as low at 2 CFU person^–1^. In the event of mussels being environmentally exposed to *E. coli* O157 and then depurated, removal of total *E. coli* spp. to end-point levels of 230 CFU 100 g^–1^ could result in residual *E. coli* O157 contamination at dangerous levels in the shellfish, despite passing end-product testing based on current bacteriological criteria. Such a case has been reported in oysters in France (Guyon et al., 2000).

### Norovirus Depuration From Mussels

At the initial stages of depuration, norovirus concentrations appeared to increase slightly in the mussel tissue relative to the initial level of concentration. It is likely that this is an artifact of the short accumulation period used. We suggest that this is caused by norovirus being present in the intravalvular fluid and non-digestive tissue material at the start of the depuration period. In the initial stages of depuration, norovirus particles continue to be processed by the mussels from the intravalvular fluid to the digestive tissue, where they bind. This increase in norovirus GII during early stages of depuration was observed in mussels experimentally contaminated with only norovirus and also in mussels exposed to mixed norovirus and *E. coli* contamination. Previous studies have shown that norovirus is not depurated effectively from oyster digestive tissues (Le Guyader et al., 2006; Ueki et al., 2007; Nappier et al., 2008), and that viruses are reduced at differential rates during depuration when compared with bacteria in mussel tissues (Power and Collins, 1989; De Mesquita et al., 1991). This study shows that over a 4 d depuration period, norovirus GII concentration remained similar to viral concentrations at the start of depuration. A recent study of norovirus depuration in mussels (*Mytilus galloprovincialis*) showed that low levels of norovirus GII (<10^3^ gc g^–1^) was successfully removed over a 7 days depuration period (Polo et al., 2014a). Our results show that norovirus GII was not reduced during depuration, in contrast to Polo et al. (2014b), and that a significant concentration of norovirus still remained after 4 days. The study of Polo et al. (2014b) used similar depuration conditions and concentrations (initial levels of murine NoV in shellfish, 4 × 10^5^ gc g^–1^) but used murine norovirus and *Mytilus galloprovincialis* which is different from our study. Considering the human infective NoV dose is ca. 10 intact virus particles (Hassard et al., 2017), then these levels may still represent a significant risk to human health. It should be noted, however, that a limitation of our study is that we did not measure infectivity directly. Caution is therefore required when interpreting the RT-qPCR RNA signal in an infectivity context (as a large proportion of the Norovirus particles may have been damaged and therefore not infective; Randazzo et al., 2017). In mussels exposed to mixed microbiological contamination, the period of time in the depuration phase where an increase in norovirus GII was observed was found to be shorter than in mussels exposed only to norovirus. Generally, lower levels of norovirus GII were measured in mussels also exposed to *E. coli* contamination potentially suggesting competition for binding sites in the digestive gland or that the presence of *E. coli* had stimulated the release of digestive enzymes. Similar evidence for this depression is also apparent in mammalian systems where bacteria have been shown to reduce norovirus infections, although the mechanisms involved still remain unclear (Hoang et al., 2015; Lee and Ko, 2017; Park et al., 2018).

Whilst the presence of norovirus in mussels appears to have no effect on the depuration efficiency of *E. coli* O157:H7, non-pathogenic *E. coli* was depurated less effectively in the presence of norovirus over the UK standard depuration time and the extended 96 h experimental depuration period. This contrasts with a previous study of *E. coli* in solo or mixed culture with *Vibrio cholerae* which demonstrated a higher effectiveness of *E. coli* depuration in *M. galloprovincialis* in the presence of *V. cholerae* (Marino et al., 2005). The reduction in efficiency of *E. coli* depuration in the presence of norovirus could be beneficial in measuring the effectiveness of depuration systems in which depurated shellfish are subject to end-product batch-testing. A reduction in the effectiveness of *E. coli* depuration as a result of norovirus contamination could result in a failure to reach the required end-point standards (<230 *E. coli* 100 g^–1^ shellfish flesh), rendering the product unmarketable.

Previous studies have found that *E. coli* in sewage contaminated shellfish is almost exclusively restricted to the digestive tract of the shellfish (Dore and Lees, 1995). In contrast, this study revealed that the proportion of *E. coli* O157:H7 DNA and environmentally derived *E. coli* found in digestive tissue to non-digestive tissues appear to vary according to the period of time for which the mussels have been exposed to the bacteria, or placed under depuration conditions. At the start of the exposure period, there appears to be a greater concentration of both *E. coli* strains in the non-digestive tissue than in the digestive tissue. It was hypothesized that this is due to the inclusion of intravalvular fluid in the non-digestive tissue. This includes the volume of water currently being processed by the mussel, which will contain bacteria in the same concentration as the surrounding seawater. The ratio of bacterial DNA in the digestive tissue to non-digestive tissue decreased over time. We ascribe this to high levels of potential bacteriolytic (lysozyme-like) activity in the intravalvular fluid (Allam and Paillard, 1998; Brousseau et al., 2018). However, in the presence of norovirus GII, the ratio of bacterial DNA in the digestive tissue to non-digestive tissue is reversed. In the absence of norovirus, there is initially more *E. coli* O157:H7 in the non-digestive tissue than in the digestive tissue. In the presence of norovirus, however, more of the *E. coli* O157:H7 is found in digestive tissue than non-digestive tissue. Norovirus is known to selectively bind to the digestive tissue (Le Guyader et al., 2006).

## Conclusion and Practical Implications

*E. coli* abundance in shellfish still represents the primary way of assessing the microbiological quality of shellfish in the European Union. Our study, however, provides strong evidence to show that this approach is fundamentally flawed under current depuration operating conditions. Overall, we have shown under both controlled laboratory and field conditions in UK waters (Winterbourn et al., 2016) that the use of non-pathogenic *E. coli* as an end-product bacteriological criterion cannot reliably assess the general microbiological risk posed from consuming contaminated shellfish. Further, it cannot be assumed that contaminated shellfish will be rendered safe under standard depuration conditions. While *E. coli* may be a suitable proxy for some bacteria (Marino et al., 2005), we show that it fails to capture the risk posed by some human pathogenic bacteria and viruses such as *E. coli* O157:H7 and norovirus GII. This supports previous work on longitudinal or spatial surveys of viral pathogens in shellfish and those performed in other shellfish species at the point of sale (Romalde et al., 2002; Formiga-Cruz et al., 2003; Flannery et al., 2009; Lowther et al., 2018). Further, there are almost no documented cases of *E. coli* poisoning by shellfish suggesting that new testing standards need to be introduced which better reflect the risk of contracting food poisoning. These standards need to be run alongside routine testing for fecal contamination using for example, *E. coli*. Alternatively, depuration conditions need to be altered to promote increased depuration rates (e.g., temperature, salinity, feeding regime; McLeod et al., 2017a). Considering the numerous shellfish-borne viral infections in humans (e.g., norovirus, hepatitis A/E; Boxman et al., 2016), we propose that a parallel routine viral test should be introduced alongside routine *E. coli* testing. Clearly, there is a rationale for choosing norovirus given the numerous shellfish-related disease outbreaks, however, its seasonal nature makes it a poor general indicator of viral contamination (e.g., hepatitis A/E). We therefore propose that human-specific sewage-associated viruses (e.g., adenovirus, crAssphage; Farkas et al., 2018, 2019), may offer an alternative indicator that can be tested alongside norovirus and *E. coli*. In addition, this can be combined with the F-RNA phage infectivity assays or capsid integrity assays to estimate the likelihood of infective viruses being resent (Flannery et al., 2009; Lowther et al., 2019; Walker et al., 2019). This information is vital if we are to introduce robust quantitative microbial risk assessments (QMRA) and adaptive management practices for commercial shellfisheries.

## Data Availability Statement

The raw data supporting the conclusions of this article will be made available by the authors, without undue reservation.

## Author Contributions

SM, DJ, and JM designed the research. JS, KC, and MD conducted the research. JS, KC, and DJ collected and analyzed the data. JS and DJ wrote the initial drafts of the manuscript. All authors contributed to the final version of the manuscript.

## Conflict of Interest

The authors declare that the research was conducted in the absence of any commercial or financial relationships that could be construed as a potential conflict of interest.
